# The impact of genotype calling errors on family-based studies

**DOI:** 10.1038/srep28323

**Published:** 2016-06-22

**Authors:** Qi Yan, Rui Chen, James S. Sutcliffe, Edwin H. Cook, Daniel E. Weeks, Bingshan Li, Wei Chen

**Affiliations:** 1 Division of Pulmonary Medicine, Allergy and Immunology; Department of Pediatrics, Children’s Hospital of Pittsburgh of UPMC, University of Pittsburgh, Pittsburgh, PA 15224, USA; 2 Department of Molecular Physiology & Biophysics, Vanderbilt Genetics Institute, Vanderbilt University Medical Center, Nashville, TN 37232, USA; 3 Department of Molecular Physiology & Biophysics, and Psychiatry, Vanderbilt University, Nashville, TN 37232, USA; 4 Department of Psychiatry, University of Illinois at Chicago, Chicago, IL 60608, USA; 5 Departments of Human Genetics and Department of Biostatistics, University of Pittsburgh Graduate School of Public Health, Pittsburgh, PA 152621, USA

## Abstract

Family-based sequencing studies have unique advantages in enriching rare variants, controlling population stratification, and improving genotype calling. Standard genotype calling algorithms are less likely to call rare variants correctly, often mistakenly calling heterozygotes as reference homozygotes. The consequences of such non-random errors on association tests for rare variants are unclear, particularly in transmission-based tests. In this study, we investigated the impact of genotyping errors on rare variant association tests of family-based sequence data. We performed a comprehensive analysis to study how genotype calling errors affect type I error and statistical power of transmission-based association tests using a variety of realistic parameters in family-based sequencing studies. In simulation studies, we found that biased genotype calling errors yielded not only an inflation of type I error but also a power loss of association tests. We further confirmed our observation using exome sequence data from an autism project. We concluded that non-symmetric genotype calling errors need careful consideration in the analysis of family-based sequence data and we provided practical guidance on ameliorating the test bias.

Next-generation sequencing is a powerful tool to dissect the genetic basis of complex diseases. Family-based sequencing studies have been conducted for various disorders such as autism^1^ and congenital heart disease^2^. Although methods for improving the accuracy of genotype calling continue to evolve, genotype calling errors, particularly at sites of low minor allele frequency, are inevitable due to imperfect sequencing technologies and limitations of current genotype calling algorithms^3^,^4^. Widely used pipelines for genotype calling often disagree and thus have low concordance rates^5^. It is well known that genotyping errors have considerable impact on type I error and power in association analysis^6^,^7^. Methods development for rare variant association tests has been an active research area in the past few years^8^,^9^,^10^,^11^, and several methods for family-based rare variant tests were recently proposed^12^,^13^,^14^. Systematic genotype-calling errors at rare variant sites can have adverse consequences on rare variant association tests, including both type I and II errors, because genotype calling methods are more likely to introduce non-random errors: calling heterozygotes as reference homozygotes rather than calling reference homozygotes as heterozygotes^15^,^16^. Without controlling for type I error, any discussion of power is meaningless. Standard approaches will suffer a great loss of power in association studies due to inefficient handling of such sequence data. Although recent efforts have been made to alleviate the problem in studies of unrelated individuals^17^, little is known for family-based sequencing studies, where the problem can be more severe because the genotypes of related people are jointly modeled in association methods. In this study we performed a comprehensive analysis to investigate the impact of genotype calling errors on family-based studies with various parameters. In addition, we analyzed real data from an autism spectrum disorder project. We showed that the bias is critical in association analyses and it not only inflates type I error but also reduces power of family-based association tests. We provided approaches and suggestions for how to reduce bias and false positive signals.

## Results

We investigated the impact of genotype calling errors on transmission-based tests as a function of several parameters: sequence coverage, gene length, calling algorithms, and different models of transmission-based tests^18^.

### Simulation study

**Figure 1:**
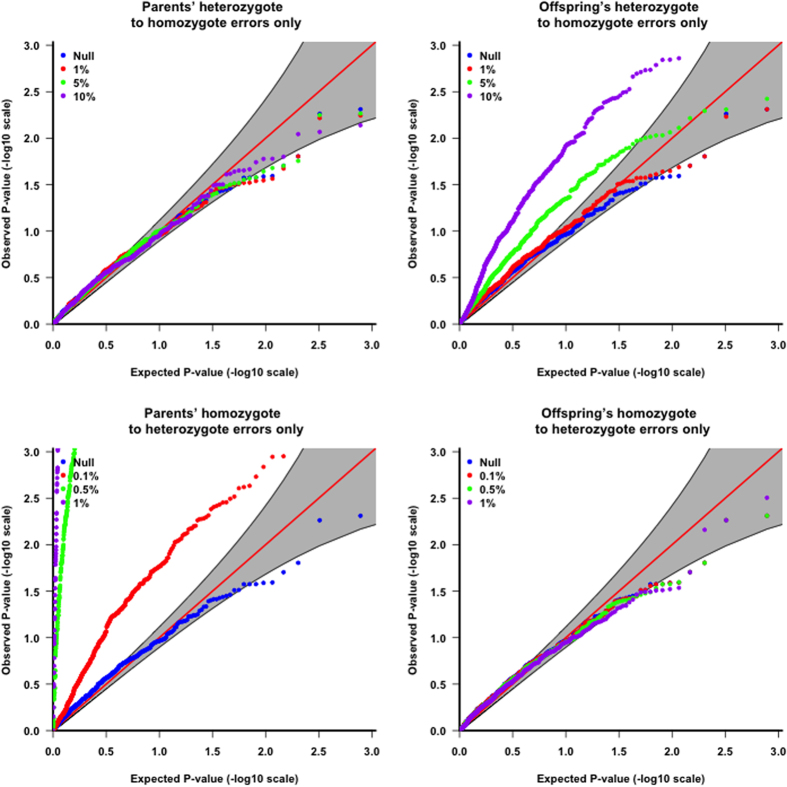
QQ plots for type I error rate simulation studies (gTDT results) with different scenarios of error patterns. We considered four scenarios to mimic this error pattern: 1. r_2_ (the error rate of calling homozygote 0/0 as heterozygote 0/1) = 0; r_1_ (the error rate of calling heterozygote 0/1 as homozygote 0/0) = 1%, 5% or 10% in parents; 2. r_2_ = 0; r_1_ = 1%, 5% or 10% in offspring; 3. r_1_ = 0; r_2_ = 0.1%, 0.5% or 1% in parents; 4. r_1_ = 0; r_2_ = 0.1%, 0.5% or 1% in offspring. The 95% point-wise confidence band (gray area) is computed under the assumption of the p-values being drawn independently from a uniform [0, 1] distribution.

We considered four scenarios described in Methods. The single marker based results (Table 1) show that the association tests could be largely influenced with the scenarios 2 (r_2_ = 0; r_1_ = 1%, 5% or 10% in offspring) and 3 (r_1_ = 0; r_2_ = 0.1%, 0.5% or 1% in parents), where r_1_ is the error rate of mistakenly calling heterozygote 0/1 as homozygote 0/0 and r_2_ is the error rate of calling homozygote 0/0 as heterozygote 0/1. For gene-level analysis, Fig. 1 shows a similar pattern with type I error rate being inflated for scenarios 2 and 3. The original transmission disequilibrium test (TDT)^19^ statistic is defined as: TDT = *(p − q)*^2^*/(p + q)*, where *p* and *q* are the counts of transmitted and non-transmitted alleles from heterozygous parents. In scenario 2 (Table 1), *p* decreases, *q* increases and *p + q* remains similar, resulting in the inflation of the TDT statistic. In scenarios 3 (Table 1), *p* remains the same and *q* increases, also resulting in inflation of the TDT statistic.

In addition to type I error rate, we studied the impact of genotype calling errors on power. Similar to that of the type I error simulation, results (Table 2) show that power of the association tests was greatly affected for scenarios 2 (r_2_ = 0; r_1_ = 1%, 5% or 10% in offspring) and 3 (r_1_ = 0; r_2_ = 0.1%, 0.5% or 1% in parents) in terms of the change of the ratio between transmitted and non-transmitted alleles. Although it is not meaningful to interpret power when type I error rate is inflated, we still show the gene-based power results (Supplementary Fig. S1) of scenario 1 that has the desired type I error rate and of scenario 2 that has inflated type I error rate. In scenario 2, as the genotyping error rate increases, the type I error rate increases and power decreases. When the genotyping error rate is greater than 5%, the type I error rate is even greater than power, which indicates that the real effect is completely canceled out by genotyping errors. We did not show the power results of scenarios 3 and 4 since the scenario of calling homozygotes as reference heterozygotes is rare in real studies and we are more interested in the scenario of calling heterozygotes as reference homozygotes. In Table 2, in scenario 2, *p* decreases, *q* increases and *p + q* remains similar, resulting in the decrease of the TDT statistic.

### Real-world study

**Figure 2:**
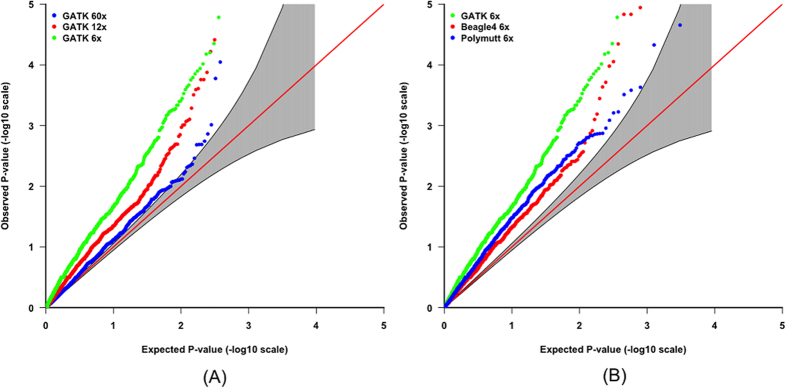
QQ plots for genes (gTDT results) in chromosome 1 from 116 parent-offspring trios from the autism study and only genotypes with GQ > 5 are used. The 95% point-wise confidence band (gray area) is computed under the assumption of the p-values being drawn independently from a uniform [0, 1] distribution. (**A**) Variant calling was carried out by GATK best-practice pipeline with different depths; (**B**) Variant calling was carried out by GATK best-practice pipeline, Beagle4 and Polymutt with the same depth of 6x.

**Figure 3:**
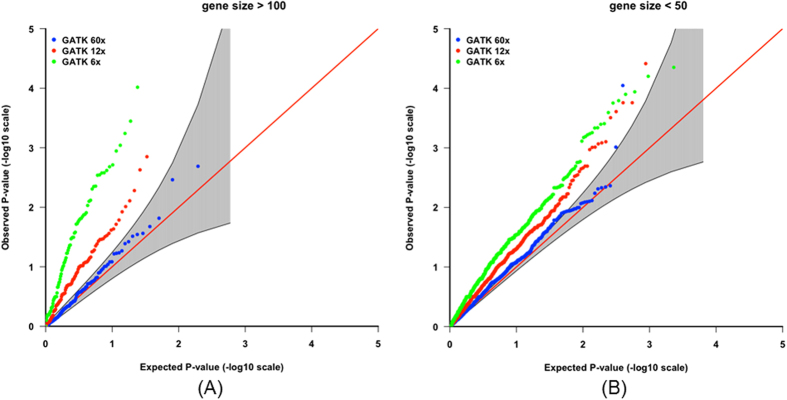
The impact of genotyping bias on different lengths of genes (gTDT results). (**A**) QQ plots for genes including more than 100 variants with different depths; (**B**) QQ plots for genes including less than 50 variants with different depths.

Results indicate that low read-depth leads to a greater reduction in the proportion of transmitted alleles (Table 3), and thus a more inflated type I error rate at the gene level (Fig. 2A). Figure 2B indicates that the Beagle4^20^ and Polymutt^21^ re-called genotypes result in reduced inflation in terms of type I error rate, but the false positive effect is still considerable. Furthermore, larger genes are more likely to be affected by genotype-calling errors compared to smaller genes, due to an accumulation of these errors (Fig. 3).

## Discussion

Genotyping error has been recognized as one of major influences on genetics association studies and investigated in various situations. This study can be viewed as a continuation of the work of Mitchell *et al*.^22^ in the context of next-generation sequencing. Mitchell *et al*. investigated the impact of genotyping errors from arrays in relatively common variants (e.g. MAF ≥ 0.01) on TDT statistics. For sequencing studies, the vast majority of variants are rare, and genotype calling is particularly challenging for rare variants. In addition, the standard analysis for rare variants is gene- or group-based strategies, which further complicates the transmission bias given potentially differential error patterns across variants in a gene or a group.

Based on both simulated and real data sets, we have assembled a comprehensive picture of how genotype calling errors impact family-based sequencing studies. Heterozygote to reference homozygote errors is by far the most common error type in rare variant calls in sequencing studies, and such errors in offspring in practice could both inflate the type I error and reduce power for transmission-based association tests. The transmission bias will be more severe for regions of low to modest coverage (30X or lower) and will be accumulated when variants are collapsed in longer genes or pathways. Standard genotype calling pipelines (e.g., GATK) do not take familial structure into account, and further refinement can be accomplished by using algorithms that do consider familial structure (e.g., Beagle4, Polymutt, or Polymutt2) to alleviate the bias.

Genotype-calling bias will not only inflate type I error but also reduce the power of subsequent association tests. Reducing power may have more detrimental effects given the inherent low power of identifying associated rare variants for complex diseases; such bias makes the rare variant association studies even more challenging. We have tried to use different methods to correct such bias, and results show that the bias can be reduced but not completely eliminated. We illustrate our findings in the design of parent-offspring trio, which is the simplest form of family structure. It will be interesting to explore this direction in future when the software for family-based rare variant association tests becomes more available. Since general pedigrees can be analyzed by treating sub-pedigrees as trios, the bias in trios can be cumulated in general pedigrees, making it a more severe problem. Although we cannot differentiate *de novo* mutation with base errors, *de novo* mutation is assumed to be extremely rare in the context of complex diseases and should not affect our conclusion. In analysis of real data, we recommend checking the direction of transmission in the top (i.e., most significant) genes to ensure that they are consistent with theoretical expectation, i.e. the fraction of genes with over-transmission are expected to be approximately 0.5 when no genes are associated with the diseases or >0.5 when genes harbor risk alleles. In situations where top ranked genes show an overall pattern of under-transmission, it may be a warning of genotype calling bias. Based on our study, and given limited resources, it may be desirable to sequence offspring at a higher coverage than parents in the up-front design of sequencing studies to mitigate such transmission bias.

## Methods

### Type I error simulation study

We simulated a set of sequence data and only retained rare variants (defined here as MAF  < 0.05) by using chromosome 22 from the 1000 Genomes Project data (see supplementary material for details). Each individual includes 182,799 SNPs across 541 genes. In each sequence data set, we simulated 100 trios with offspring as disease cases. Furthermore, we assigned errors to the sequence data set. Since the biased error rate of mistakenly calling heterozygote 0/1 as homozygote 0/0 (this error rate is denoted as r_1_) is much larger than the error rate of calling homozygote 0/0 as heterozygote 0/1 (this error rate is denoted as r_2_) for rare variants, we considered four scenarios to mimic this error pattern: 1. r_2_ = 0; r_1_ = 1%, 5% or 10% in parents; 2. r_2_ = 0; r_1_ = 1%, 5% or 10% in offspring; 3. r_1_ = 0; r_2_ = 0.1%, 0.5% or 1% in parents; 4. r_1_ = 0; r_2_ = 0.1%, 0.5% or 1% in offspring. Although we assumed the error rate of 0.1%, 0.5% and 1% for the scenarios 3 and 4 in the simulations, the occurrence of these two types of error is extremely low in reality due to the nature of genotype calling strategy^23^. The scenarios 1 and 2 represent the majority of errors in real studies. The allele frequency distribution of simulated genotype data sets for this type I error rate study is shown in Supplementary Fig. S2. To study the impact of these different scenarios on the transmission-based association methods, we first applied the widely used transmission disequilibrium test (TDT)^19^ implemented in PLINK^24^ on each of the SNPs to calculate the total number of alleles that are transmitted or not transmitted from parents to offspring. Because single marker tests are known to be less powerful to detect rare variant associations, rare variants are usually grouped into genes and tested at the gene level^25^,^26^,^27^,^28^. We used the gTDT (http://genome.sph.umich.edu/wiki/GTDT) that can be viewed as an extension of TDT for a gene-based analysis^18^. The genotyping errors can introduce inconsistencies (i.e., Mendelian errors) in the trios and these inconsistent trios are excluded in TDT and gTDT.

### Power simulation study

We simulated a set of sequence data of 100 trios and 1,000 genes that contain 19,103 rare variants (MAF < 0.01). We randomly assigned the effect size β = log(4) to 30% of the variants. The power simulation details are described elsewhere^18^. Briefly, we generated genotypes of parents based on allele frequencies and randomly transmitted one haplotype from each of the parents to their offspring to simulate a parent-office trio. The offspring was designated as affected according to the probability of being diseased based on the effect sizes of the casual variants. The allele frequency distribution of simulated genotype data sets for this power study is shown in Supplementary Fig. S3.

### Real-world study

We obtained exome sequence data from a trio study of autism spectrum disorder (ASD). Details of the data are described previously^18^,^29^. The high coverage of the data (~60X) allows us to investigate impact of sequencing coverage using downsampling. We used chromosome 1 sequence data from 116 parent-offspring trios for this investigation. A subset of the reads was extracted to construct a set of data with depth of 6X and 12X for comparison purposes. Variant calling was carried out using the GATK 3.3.0 best-practice pipeline. Each individual includes 74,652 overlapped rare SNPs (MAF < 0.05) in both data sets, which can be mapped to 2,283 genes. Similar to the above simulations, we used the TDT test to calculate transmitted and non-transmitted alleles for single SNPs and the gTDT in gene-based tests to investigate inflation caused by genotype calling errors. Because GATK does not take familial correlations into account, it leads to lower accuracy of calls, especially for low depth sites (e.g. 6X). Therefore, we applied two existing family-based genotype-calling methods, Beagle4^20^ and Polymutt^21^, to re-call the genotypes at sites with depth of 6X.

## Additional Information

**How to cite this article**: Yan, Q. *et al*. The impact of genotype calling errors on family-based studies. *Sci. Rep.* **6**, 28323; doi: 10.1038/srep28323 (2016).

## Supplementary Material

Supplementary Information

## Figures and Tables

**Table 1:** The total transmitted and non-transmitted alleles over all 182,799 SNPs for single SNP TDT test in type I error rate simulation studies (each SNP could have none or multiple transmitted and non-transmitted alleles).

	Null	r2 = 0; r1 = 1% Parents	r2 = 0; r1 = 5% Parents	r2 = 0; r1 = 10% Parents
Transmitted	374,502 (47%)	370,753 (47%)	355,759 (47%)	337,142 (47%)
Non-transmitted	427,972 (53%)	423,684 (53%)	406,135 (53%)	384,324 (53%)
		r2 = 0; r1 = 1% Offspring	r2 = 0; r1 = 5% Offspring	r2 = 0; r1 = 10% Offspring
Transmitted		370,880 (46%)	356,113 (44%)	338,061 (42%)
Non-transmitted		431,594 (54%)	446,354 (56%)	464,397 (58%)
		r1 = 0; r2 = 0.1% Parents	r1 = 0; r2 = 0.5% Parents	r1 = 0; r2 = 1% Parents
Transmitted		374,502 (45%)	374,502 (38%)	374,502 (32%)
Non-transmitted		463,784 (55%)	607,270 (62%)	785,472 (68%)
		r1 = 0; r2 = 0.1% Offspring	r1 = 0; r2 = 0.5% Offspring	r1 = 0; r2 = 1% Offspring
Transmitted		374,912 (47%)	376,561 (47%)	378,642 (47%)
Non-transmitted		427,562 (53%)	425,913 (53%)	423,832 (53%)

**Table 2:** The total transmitted and non-transmitted alleles over all 19,103 SNPs for single SNP TDT test in power simulation studies (each SNP could have none or multiple transmitted and non-transmitted alleles).

	Original	r2 = 0; r1 = 1% Parents	r2 = 0; r1 = 5% Parents	r2 = 0; r1 = 10% Parents
Transmitted	17,225 (61%)	17,050 (61%)	16,361 (61%)	15,463 (61%)
Non-transmitted	11,112 (39%)	11,000 (39%)	10,518 (39%)	9,981 (39%)
		r2 = 0; r1 = 1% Offspring	r2 = 0; r1 = 5% Offspring	r2 = 0; r1 = 10% Offspring
Transmitted		17,032 (60%)	16,349 (58%)	15,559 (55%)
Non-transmitted		11,305 (40%)	11,988 (42%)	12,778 (45%)
		r1 = 0; r2 = 0.1% Parents	r1 = 0; r2 = 0.5% Parents	r1 = 0; r2 = 1% Parents
Transmitted		17,225 (54%)	17,225 (36%)	17,224 (26%)
Non-transmitted		14,938 (46%)	30,096 (64%)	48,833 (74%)
		r1 = 0; r2 = 0.1% Offspring	r1 = 0; r2 = 0.5% Offspring	r1 = 0; r2 = 1% Offspring
Transmitted		17,236 (61%)	17,817 (63%)	17,349 (61%)
Non-transmitted		11,101 (39%)	10,520 (37%)	10,988 (39%)

**Table 3:** The total transmitted and non-transmitted alleles for single SNP TDT test in chromosome 1 from 116 parent-offspring trios from the autism study.

	60x	12x	6x
Transmitted	108,467 (47%)	48,769 (40%)	19,454 (32%)
Non-transmitted	124,184 (53%)	72,287 (60%)	41,744 (68%)
