# Supplementary material for: The impact of genotype calling errors on family-based studies
Source: Sci Rep. 2016 Jun 22;6:28323. doi: 10.1038/srep28323 (PMC4916415; doi:10.1038/srep28323)
Supplement: Supplementary Information [file srep28323-s1.pdf]

The impact of genotype calling errors on family-based studies

Qi Yan[1], Rui Chen[2], James S. Sutcliffe[3], Edwin H. Cook[4], Daniel E. Weeks[5], Bingshan Li[2*], Wei Chen[1,5*]

[1]Division of Pulmonary Medicine, Allergy and Immunology; Department of Pediatrics, Children's Hospital of Pittsburgh of UPMC, University of Pittsburgh, Pittsburgh, PA 15224, USA

[2]Department of Molecular Physiology & Biophysics, Vanderbilt Genetics Institute, Vanderbilt University Medical Center, Nashville, TN 37232, USA

[3]Department of Molecular Physiology & Biophysics, and Psychiatry, Vanderbilt University, Nashville, TN 37232, USA

[4]Department of Psychiatry, University of Illinois at Chicago, Chicago, IL 60608, USA

[5]Departments of Human Genetics and Biostatistics, University of Pittsburgh Graduate School of Public Health, Pittsburgh, PA 152621, USA

[*] Correspondence: wei.chen@chp.edu; bingshan.li@vanderbilt.edu

Supplemental Material

Simulation of sample genotypes for type I error rate: We simulated sample genotypes based on a pool of 2,184 haplotypes (i.e., 1,092 samples) over chromosome 22 from 1000 Genomes Project data. We simulated 100 trio families with father, mother and one child by first randomly selecting 400 haplotypes as the parents' haplotypes. The offspring haplotypes were generated by randomly transmitting one of the two haplotypes of the father and the mother to the child. Finally, we simulated 100 trios. Only the rare variants (defined as $MAF<0.05$) were retained.

Figures

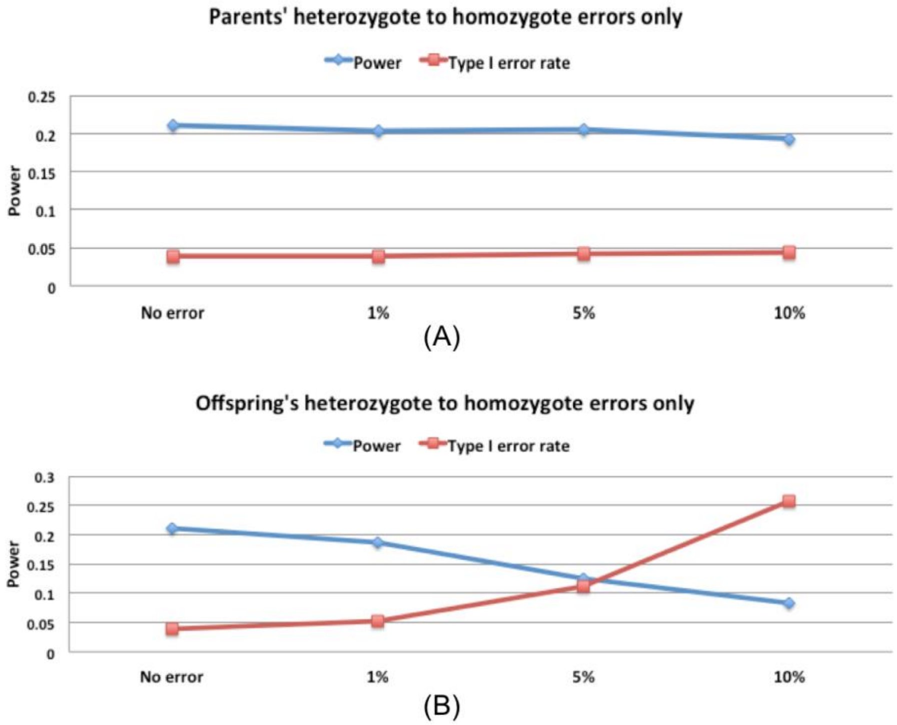

Figure S1. Power simulation results for gTDT at α=0.05. The blue lines are the power and the red lines are the type I error rate for the corresponding scenarios. (A)The scenario 1 (r_2=0; r_1=1%, 5% or 10% in parents). (B) The scenario 2 (r_2=0; r_1=1%, 5% or 10% in offspring).

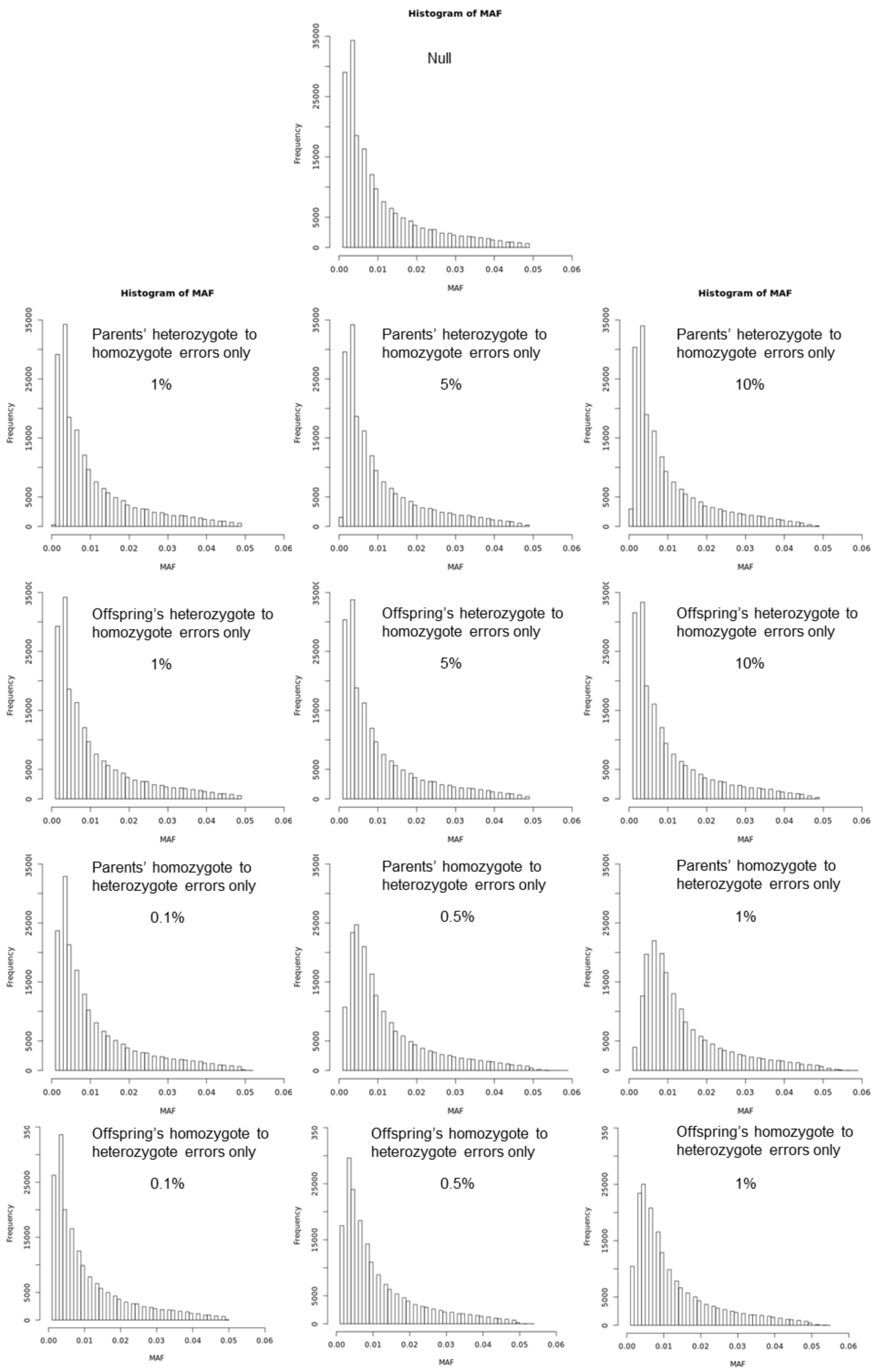

Figure S2. Distribution of simulated genotype data sets for type I error rate study. The *x*-axis shows the SNP minor allele frequencies (MAF) and *y*-axis shows the SNP counts.

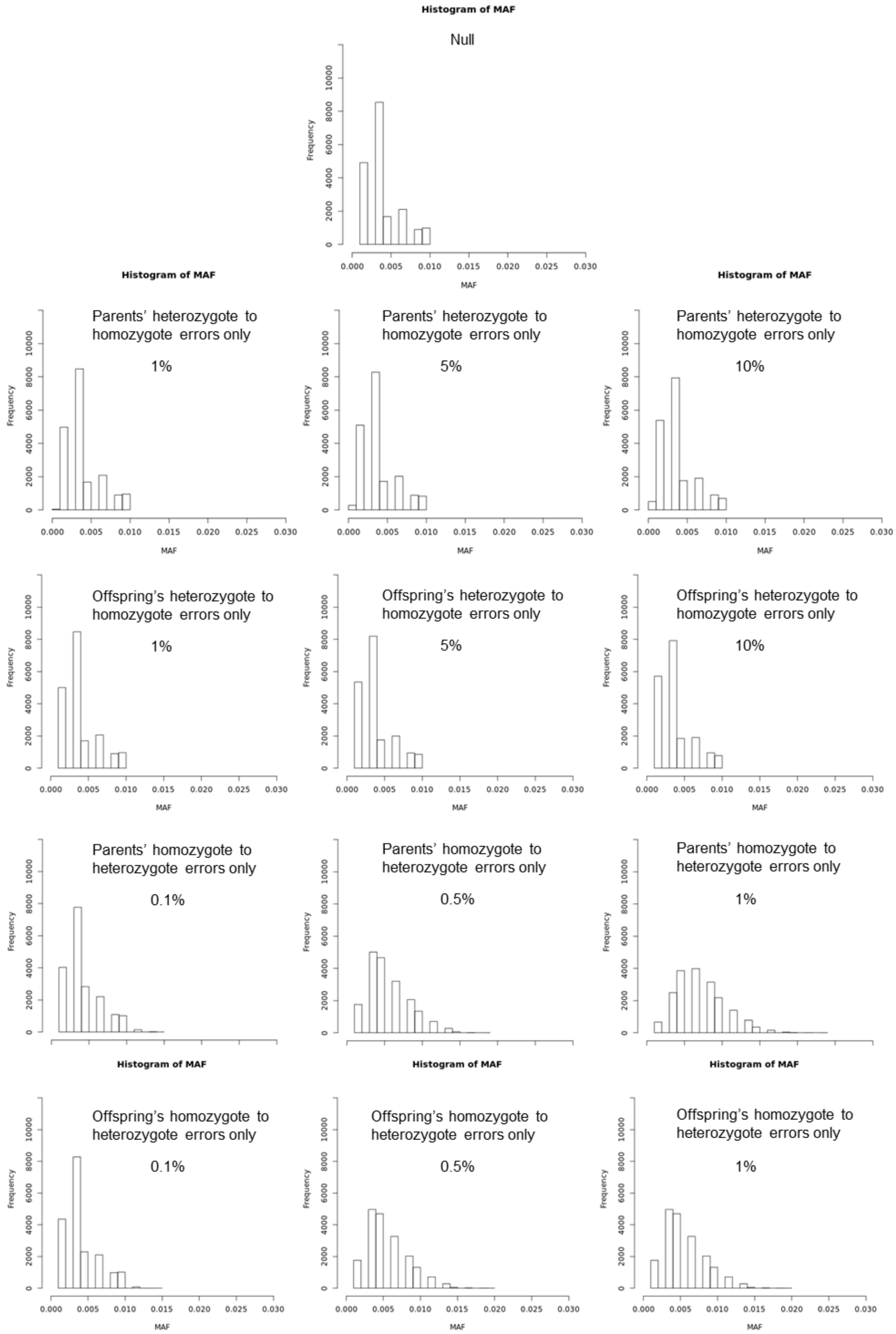

Figure S3. Distribution of simulated genotype data sets for power study. The *x*-axis shows the SNP minor allele frequencies (MAF) and *y*-axis shows the SNP counts.
